# The complete mitochondrial genome of the firefly, *Pteroptyx maipo* (Coleoptera: Lampyridae)

**DOI:** 10.1080/23802359.2017.1398598

**Published:** 2017-11-08

**Authors:** Yadong Fan, Xinhua Fu

**Affiliations:** a Hubei Insect Resources Utilization and Sustainable Pest Management Key Laboratory, College of Plant Science and Technology, Huazhong Agricultural University, Wuhan, Hubei, China;; b Firefly Conservation Research Centre, Wuhan, Hubei, China

**Keywords:** *Pteroptyx maipo*, firefly, lampyridae, mitochondrial genome

## Abstract

We report the complete mitochondrial genome of firefly, *Pteroptyx maipo*. The circular genome of 16,127 bp has a base composition of A (44.22%), C (11.60%), G (8.23%) and T (35.96%). Our sequence is similar to other Metazoa, which contains 13 protein-coding genes. All 13 protein-coding genes were initiated by the ATN (ATT, ATA and ATG) codon. Nine protein-coding genes stopped with TAA or TAG codon and the other four genes have an incomplete termination codon, a single T. We sequenced the mitochondrial genome of fireflies to analyze phylogenetic relationships and deduce the evolution of their flashing signals.

## Introduction

Firefly are a family of insects in the beetle order Coleoptera. Using morphological characters, 30 species are recognised in the genus *Pteroptyx* following revisions by Ballantyne and McLean (Ballantyne and McLean 1970; Ballantyne 1987a, 1987b, 2001). Some *Pteroptyx* species have spectacular synchronous flashing behavior (Lloyd 1973; Case 1980; Copeland and Moiseff 1997, 2004; Jusoh et al. 2013). *Pteroptyx maipo* Ballantyne et al. (2011) is the first species of *Pteroptyx* firefly recorded in China and distributed in Hong Kong (Ballantyne et al. 2011). *Pteroptyx maipo* flash like other Luciolinae fireflies but not in synchrony (Ballantyne et al. 2011).

Mitochondrial genome sequences are essential to a comprehensive understanding of the evolution of Lampyridae and other luminescent beetles (Ermakov et al. 2006). Here, we elucidate the mtDNA genome of *P. maipo*.

These male fireflies used in this study were collected from Enping city, Guangdong Province, China (22°01′N, 112°21′E) in 13 July 2013, and were stored in Natural History Museum, Huazhong Agricultural University, Wuhan, Hubei, China (its accession no. PM2011052301). *Pteroptyx maipo*’s habits, flashing signals and some morphology have been studied in detail (Ballantyne et al. 2011). However, there is no genetic research information about *P. maipo.*

Specific primers were designed based on these conserved regions sequences. The PCR reaction was carried out with LA Taq polymerase for 35 cycles at 94 °C for 30 s, and annealed at 50 °C for 30 s, followed by extension at 72 °C for 1 min per 1 kb. Sequences were assembled using the software DNAstar v7.1 (DNAstar, Madison, WI) and adjusted manually to generate the complete sequence of mitochondrial DNA.

The complete mitochondrial genome sequence of *P. maipo* (GenBank MF686051) has 16,127 bp and has a base composition of A (44.22%), C (11.59%), G (8.23%), and T (35.96%). Similar to other Metazoa, our sequence contains 13 protein-coding genes, 22 transfer RNA genes, two ribosomal RNA genes and a non-coding AT-rich region, which represents a typical insect mitochondrial genome (Wolstenholme 1992). The open frames of the 13 protein-coding genes were inferred from three other fireflies: *Aquatica leii*, *Luciola substriata,* and *Pyrocoelia rufa* (Lee et al. 2004; Jiao et al. 2015; Mu et al. 2016). All 13 PGGs initiated with ATN (ATT, ATA, and ATG) codon. Among those genes, six PCGs (COII, ATP6, COIII, ND4, ND4L, and CYTB) initiate from ATG, and five PCGs (COI, ATP8, ND5, ND6, and ND1) initiate from ATT, and two PCGs (ND2 and ND3) start with ATA. Six PCGs are terminated with TAA (ND2, COI, ATP8, ATP6, ND4L, and ND6). Three are ended with TAG (ND3, CYTB, and ND1). And four PCGs (COII, COIII, ND5, and ND4) terminate with incomplete stop codon T. The 12S and 16S ribosomal RNA genes are 743 and 1264 bp, respectively. The length of the 22 tRNA genes ranged from 62 to 71 bp. The AT-rich region is 1550 bp.

**Figure 1.**
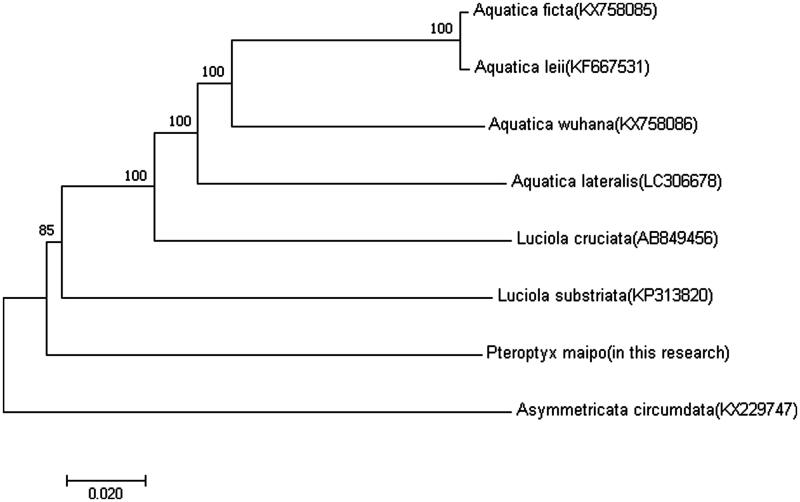
Molecular phylogeny of *Pteroptyx maipo* and seven other firefly species based on the complete mitochondrial genome. The complete mitochondrial genome was downloaded from GenBank and the phylogenic tree was constructed by neighbour-joining method with 1000 bootstrap replicates. MtDNA accession numbers used for tree construction are as follows: *Aquatica ficta* (KX758085), *Aquatica leii* (KF667531), *Aquatica wuhana* (KX758086), *Luciola cruciata* (AB849456), *Asymmetricata circumdata* (KX229747), *Aquatica lateralis* (LC306678), and *Luciola substriata* (recently identified as *Sclerotia flavida* by Ballantyne et al. 2016) (KP313820).

The phylogenetic tree among the five species based on mitochondrial genome sequences were aligned in MEGA 5 (Phoenix, AZ) (with 1000 bootstrap replicates) to construct a Neighbour-Joining tree (Figure 1).

The result shows *P. maipo* is most closely related to *Luciola Substriata* (recently identified as *Sclerotia flavida* by Ballantyne et al. 2016), which belongs to an entirely different genus in the Lampyridae.

In conclusion, the complete mitochondrial genome sequence of *P. maipo* provides an important molecular framework for further phylogenetic analyses of fireflies. These data are essential for comprehensive understanding of the role of sexual and natural selection in the evolution of firefly flashing signals.
